# Medical radiation exposure during gastrointestinal enteral metallic stent placement: Post hoc analysis of the REX‐GI study

**DOI:** 10.1002/jgh3.12993

**Published:** 2023-12-01

**Authors:** Junki Tokura, Toshiyuki Yoshio, Shiro Hayashi, Masashi Yamamoto, Satoshi Asai, Takayuki Yakushijin, Kenji Ikezawa, Koji Nagaike, Tadayuki Takagi, Toshio Fujisawa, Takuya Yamada, Hidetaka Tsumura, Iruru Maetani, Yasuki Hori, Hideyuki Ihara, Kazuhiro Matsunaga, Toshio Kuwai, Yukiko Ito, Kenkei Hasatani, Yoriaki Komeda, Akira Kurita, Shinjiro Yamaguchi, Hirotsugu Maruyama, Takuji Iwashita, Mamoru Takenaka, Makoto Hosono, Tsutomu Nishida

**Affiliations:** ^1^ Department of Gastroenterology Cancer Institute Hospital, Japanese Foundation for Cancer Research Tokyo Japan; ^2^ Department of Gastroenterology and Internal Medicine Hayashi Clinic Suita Japan; ^3^ Department of Gastroenterology Toyonaka Municipal Hospital Toyonaka Japan; ^4^ Department of Gastroenterology Tane General Hospital Osaka Japan; ^5^ Department of Gastroenterology and Hepatology Osaka General Medical Center Osaka Japan; ^6^ Department of Hepatobiliary and Pancreatic Oncology Osaka International Cancer Institute Osaka Japan; ^7^ Department of Gastroenterology and Hepatology Suita Municipal Hospital Osaka Japan; ^8^ Department of Gastroenterology, Fukushima Medical University School of Medicine Fukushima Japan; ^9^ Department of Gastroenterology Graduate School of Medicine, Juntendo University Tokyo Japan; ^10^ Department of Gastroenterology and Hepatology Osaka Rosai Hospital Sakai Japan; ^11^ Department of Gastroenterological Oncology Hyogo Cancer Center Akashi Japan; ^12^ Division of Gastroenterology and Hepatology, Department of Internal Medicine Toho University Ohashi Medical Center Tokyo Japan; ^13^ Department of Gastroenterology and Metabolism Nagoya City University Graduate School of Medical Sciences Nagoya Japan; ^14^ Department of Gastroenterology Tonan Hospital Sapporo Japan; ^15^ Department of Gastroenterology Ishikawa Prefectural Central Hospital Kanazawa Japan; ^16^ Department of Gastroenterology, National Hospital Organization Kure Medical Center and Chugoku Cancer Center Kure Japan; ^17^ Department of Gastroenterology Japanese Red Cross Medical Center Tokyo Japan; ^18^ Department of Gastroenterology Fukui Prefectural Hospital Fukui Japan; ^19^ Department of Gastroenterology and Hepatology, Faculty of Medicine Kindai University Ōsakasayama Japan; ^20^ Department of Gastroenterology and Hepatology Digestive Disease Center, Kitano Hospital, Tazuke Kofukai Medical Research Institute Osaka Japan; ^21^ Department of Gastroenterology and Hepatology Kansai Rosai Hospital Amagasaki Japan; ^22^ Department of Gastroenterology Osaka City University Graduate School of Medicine Osaka Japan; ^23^ First Department of Internal Medicine Gifu University Hospital Gifu Japan

**Keywords:** diagnostic reference level, enteral metallic stent placement, fluoroscopy, gastrointestinal stenosis, radiation exposure

## Abstract

**Background and Aim:**

Recently, the use of various endoscopic procedures performed under X‐ray fluoroscopy guidance has increased. With the popularization of such procedures, diagnostic reference levels (DRLs) have been widely accepted as the global standard for various procedures with ionizing radiation. The Radiation Exposure from Gastrointestinal Fluoroscopic Procedures (REX‐GI) study aimed to prospectively collect actual radiation exposure (RE) data and establish DRLs in gastrointestinal endoscopy units. In this post hoc analysis of the REX‐GI study, we established DRLs for each disease site by analyzing cases of gastrointestinal enteral metallic stent placement.

**Methods:**

The REX‐GI study was a multicenter, prospective observational study conducted to collect actual RE data during gastrointestinal enteral metallic stent placement. To establish DRL values for three disease sites, namely the esophagus, gastroduodenum, and colon, we examined fluoroscopy time (FT; min), number of X‐ray images, air kerma at the patient entrance reference point (*K*_a,r_; mGy), and the air kerma–area product (*P*_KA_; Gy cm^2^) during enteral metallic stent placement.

**Results:**

Five‐hundred and twenty‐three stenting procedures were performed. The DRL values of FT (min) and the number of X‐ray images for the esophagus/gastroduodenum/colon were 9/16/18 min and 9/15/11 min, respectively. Furthermore, the DRL values of *K*_a,r_ and *P*_KA_ for each disease site were 43.3/120/124 mGy and 10.3/36.6/48.4 Gy cm^2^, respectively. Among the procedures, esophageal stents were significantly associated with the lowest values (*P* < 0.001).

**Conclusion:**

The characteristics of RE vary according to disease site among gastrointestinal enteral metallic stent placements. Thus, it is desirable to set DRL values based on the disease site.

## Graphical abstract

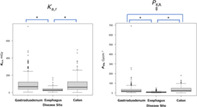

## Introduction

Medical radiation is widely used not only in medical imaging but also in radiation treatment. In medical imaging, fluoroscopy uses radiation to visualize a continuous X‐ray image on a monitor and plays a significant role in the daily practices of gastroenterology, including digestive endoscopic as well as hepatobiliary and pancreatic studies. Radiological imaging has both benefits and disadvantages for the patients. The latter are further divided into two types: deterministic risks,^1^ determined by the threshold dose, as represented by skin injury; and stochastic risks, determined by a linear no‐threshold model, such as cancer risk.^2^ The optimization of patient protection from radiation exposure (RE) from diagnostic and interventional procedures requires the application of examination‐specific protocols. The International Commission on Radiological Protection (ICRP) and the International Atomic Energy Agency (IAEA) have introduced diagnostic reference levels (DRLs) to optimize protection against RE due to medical imaging with ionizing radiation.^3^ The DRL values are usually set at the 75th percentile of a typical sample dose distribution.^4^ The ICRP recommends considering DRLs as much as possible during all procedures using radiation because the cumulative fluoroscopy exposure time is a poor metric of patient radiation dose. DRLs are now broadly accepted as the global standard for all procedures with ionizing radiation and have been established in Japan (Japan DRLs 2015, updated in 2020).^5^, ^6^ However, these DRLs still require further optimization for specific procedures, particularly fluoroscopy‐guided gastrointestinal procedures.

Enteral metallic stent placement for malignant gastrointestinal obstruction has been widely used in recent years. Since the development of esophageal stents for esophageal obstruction, gastroduodenal stents and colonic stents that can be implanted through an endoscope have become available in daily practice.^7^, ^8^, ^9^, ^10^, ^11^, ^12^, ^13^ The treatment outcomes of cancers that cause gastrointestinal stenosis are also continuously improving, and the frequency of malignant gastrointestinal stenosis is increasing as patient survival is prolonged. Enteral metallic stent placement is performed in various organs depending on the disease site. Moreover, the type of stents used and the difficulty of the procedure are all quite different among the disease sites. Thus, DRLs for enteral metallic stent placement are not sufficient because of the lack of specific DRLs for different disease sites.^14^ In the present study, we aimed to determine DRLs for each disease site by analyzing cases of gastrointestinal enteral metallic stent placement from the Radiation EXposure from GastroIntestinal Fluoroscopic Procedures (REX‐GI) study, which is a nationwide, prospective observational study.

## Material and methods

### Study design

This study is a post hoc analysis of a multicenter, prospective observational cohort study that collected real radiation dose data from ERCP, interventional EU, balloon‐assisted enteroscopy, enteral metallic stent placement, and enteral tube placement from May 2019 to December 2020. Overall, 12 959 fluoroscopy‐guided gastrointestinal procedures were included from 23 hospitals in Japan, including 8 university hospitals, 4 cancer centers, 9 general hospitals, and 2 municipal hospitals; we focused on enteral metallic stent placement in this post hoc analysis, which was scheduled in the original protocol.^15^ The rationale and methodology of the study have been published, and the full protocol is available online.^14^

### Study population

We included consecutive patients receiving standard clinical care who underwent enteral metallic stent placement. The study period was between May 2019 and December 2020. We examined the fluoroscopy time (FT; min), the number of X‐ray images, air kerma at the patient entrance reference point (*K*_a,r_; mGy), and air kerma–area product (*P*_KA_; Gy cm^2^) during enteral metallic stent placement. Air kerma is the dose defined on the basis of the amount of energy received per unit mass of air exposed to X‐rays; *K*_a,r_ is the air kerma at the patient entrance reference point accumulated throughout the X‐ray procedure; *P*_KA_ is the integral of the air kerma (Gy) in the irradiated field and is expressed in units of Gy cm^2^.

We also calculated the respective values at the disease site. The procedure time (PT) of enteral metallic stent placement for three disease sites, namely the esophagus, gastroduodenum, and colon, was examined. These data were collected from the fluoroscopy equipment at each institution. The facility representative sent these data to the data center every 3 months throughout the study period. Patients who indicated that they did not want to participate in this study via the opt‐out method on the website of each hospital, patients with multiple missing primary outcomes (e.g., *K*_a,r_, and *P*_KA_), and duplicate enrollments were considered unsuitable for inclusion in this study. These patients were excluded by two of the authors (HS and NT). Although it is necessary to specify the participating institutions, we have purposely not shown the data for each institution to maintain the anonymity of the institutions.

### Data analysis

Continuous variables are reported as the median and third quartile. Categorical variables are expressed as numbers or frequencies. DRLs were set at the 75th percentile of the distribution of each sample dose. Data analysis was performed by using a comparative Mann–Whitney *U* test and the Kruskal–Wallis test. All statistical analyses were performed with JMP software (SAS Institute, Cary, NC, USA).

## Results

### Patient and hospital characteristics

The characteristics of the patients and hospitals are shown in Table 1. A total of 523 stenting procedures were performed. The median age of the patients was 72 years, and 311 patients were male (58.3%). The disease sites were the esophagus, gastroduodenum, large intestine, and jejunum in 114 (21.8%), 262 (50.1%), 143 (27.3%), and 4 (0.7%) cases, respectively.

**Table 1:** Patient and hospital characteristics

Total number of patients	523
Male, *n*, %	311 (58.3)
Age, median (IQR)	72 (65–81)
Type of fluoroscopy equipment,^†^ overtype: number, *n*, %	15 (65%)
Year of fluoroscopy equipment,^†^ median (range)	2016 (2004–2019)
Basic setting^‡^ of the irradiated field (cm^2^): median (IQR)	25 (21–42)
Basic setting^‡^ of the frame rate (frames per second): median (IQR)	12.5 (7.5–15)
Fluoroscopy operator, outside: number (%)	14 (61%)
Disease site	
Esophagus, *n* (%)	114 (21.8)
Gastroduodenum, *n* (%)	262 (50.1)
Colon, *n* (%)	143 (27.3)
Jejunum, *n* (%)	4 (0.8)

^†^ The main unit was registered if a hospital had multiple fluoroscopy units.

^‡^ “Basic setting” means the setting of the fluoroscopy unit at the start of the procedure; it does not reflect any changes made during the procedure.

IQR, interquartile range.

Regarding the fluoroscopy equipment in the institutions, 15 out of 23 facilities (65%) used the overtube type, and 14 (61%) had fluoroscopic equipment operated externally. The median year of introduction of fluoroscopy equipment in all hospitals was 2016. The basic setting of the median irradiated field was 25 cm^2^, and the median frame rate was 12.5 fps.

### Fluoroscopy time and the number of X‐ray images for each disease site

**Figure 1:**
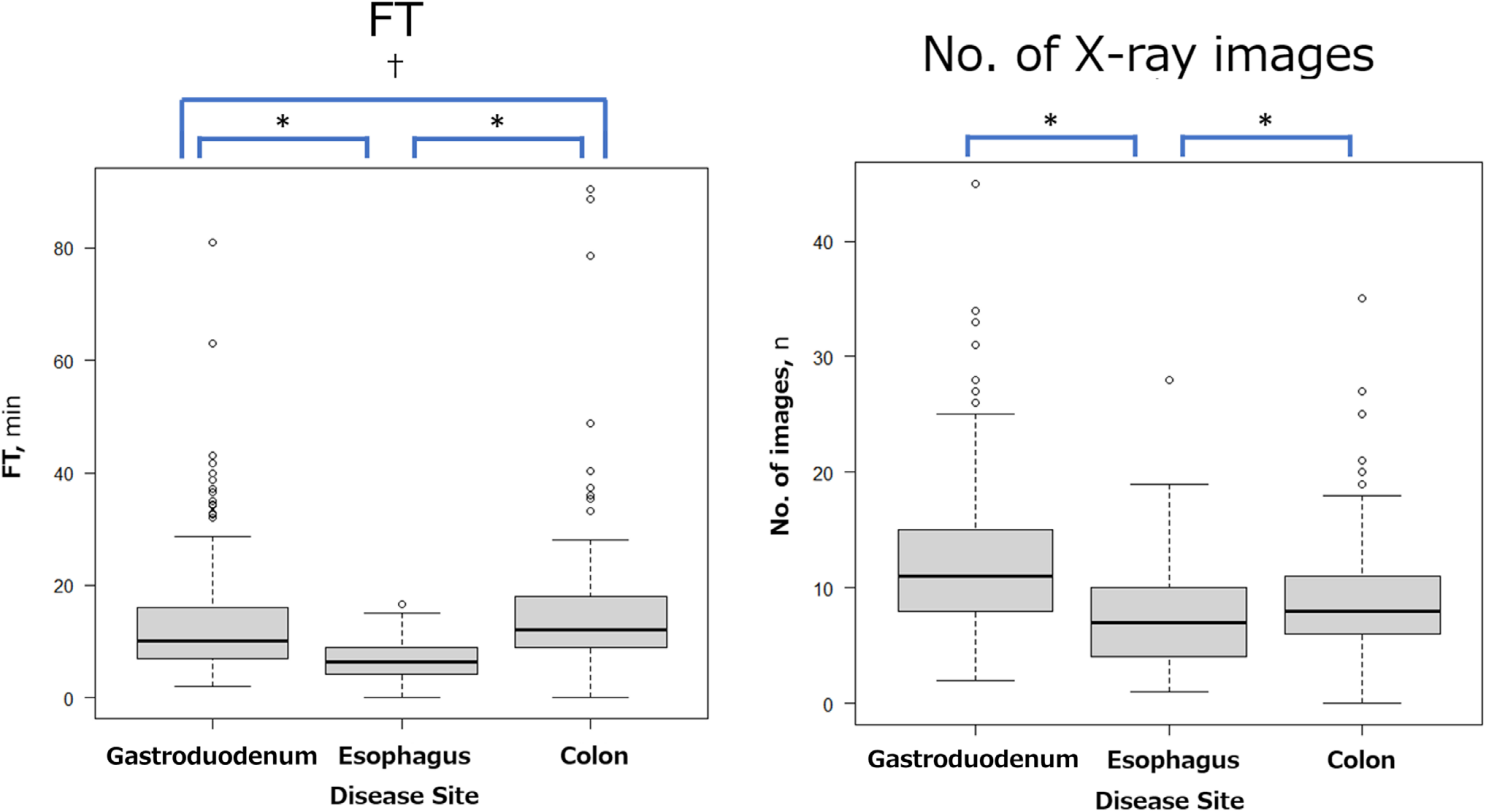
Comparison of each disease site for fluoroscopy time (FT: min) and the number of X‐ray images during enteral metallic stent placement. Esophageal stenting had a significantly shorter FT than gastroduodenal and colonic stenting, and gastroduodenal stenting had a shorter FT than colonic stenting. For the number of images, the median value was lowest for esophageal stenting. *P < 0.001; ^†^ *P* = 0.0248.

The median and third quartile values of FT (min), as well as the number of X‐ray images for each disease site, are shown in Table 2. For each disease site (esophagus, gastroduodenum, and colon), FT and the number of X‐ray images per examination were analyzed. For the esophagus, the median/third quartile values of FT and the number of images were 6.4/9 min and 7/9, respectively. Similarly, these values were 10.1/16 min and 11/15 for the gastroduodenum and 12/18 min and 8/11 for the colon, respectively. For the jejunum, these values were not compiled because the number of cases was small. A comparison of these values for each disease site is shown in Figure 1. Esophageal stenting had a significantly shorter FT than gastroduodenal and colonic stenting (*P* < 0.001), and gastroduodenal stenting had a shorter FT than colonic stenting (*P* = 0.0248). For the number of images, the median value was lowest for esophageal stenting (*P* < 0.001).

**Table 2:** Median and third quartile values of FT, number of X‐ray images, *K*_a,r_, and *P*_KA_ for each disease site

	Disease site		
	Esophagus	Gastroduodenum	Colon
FT (min)	6.4, 9	10.1, 16	12, 18
Median, third quartile			
No. of X‐ray images	7, 9	11, 15	8, 11
Median, third quartile			
*K*_a,r_ (mGy)	27.7, 43.3	66.8, 120	61, 124
Median, third quartile			
*P*_KA_ (Gy cm^2^)	6.7, 10.3	19, 36.6	22.4, 48.4
Median, third quartile			

FT, fluoroscopy time; *K*_a,r_, air kerma at the patient entrance reference point; *P*_KA_, air kerma–area product.

### *K* _a,r,_ and *P*_KA_ for each disease site

**Figure 2:**
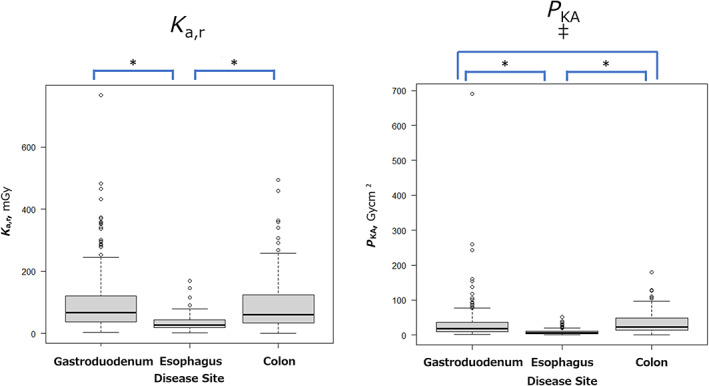
Comparison of each disease site for air kerma at the patient entrance reference point (*K*_a,r_; mGy) and air kerma–area product (*P*_KA_; Gy cm^2^). For *K*_a,r_, a comparison of the median values showed that esophageal stenting had a significantly lower value than gastroduodenal and colonic stenting. For *P*_KA_, esophageal stenting had a significantly lower value than gastroduodenal and colonic stenting, and the value for gastroduodenal stenting tended to be lower than that for colonic stenting (*P* = 0.0759). *P < 0.001; ^‡^ *P* = 0.0759.

The median and third quartile values of *K*_a,r_ (mGy) and *P*_KA_ (Gy cm^2^) for each disease site are shown in Table 2. For each disease site, *K*_a,r_ and *P*_KA_ per examination were analyzed. For the esophagus, the median/third quartile values of *K*_a,r_ and *P*_KA_ were 27.7/43.3 mGy and 6.7/10.3 Gy cm^2^, respectively. Similarly, for the gastroduodenum, these values were 66.8/120 mGy and 19/36.6 Gy cm^2^, respectively, and for the colon they were 61/124 mGy and 22.4/48.4 Gy cm^2^, respectively. A comparison of these values for each disease site is shown in Figure 2. For *K*_a,r_, a comparison of the median values showed that esophageal stenting had a significantly lower value than gastroduodenal and colonic stenting (*P* < 0.001), whereas there was no difference between gastroduodenal and colonic stenting (*P* = 1.000). Similarly, for *P*_KA_, esophageal stenting had a significantly lower value than gastroduodenal and colonic stenting (*P* < 0.001), and the value for gastroduodenal stenting tended to be lower than that for colonic stenting (*P* = 0.0759).

### PT for stenting for each disease site

The median PT for stenting at each disease site was 21 min for the gastroduodenum, 16 min for the esophagus, and 24.5 min for the colon. The PT for esophageal stenting was significantly shorter than that for other disease sites (*P* < 0.001). PT for colonic stenting was significantly longer than that for gastroduodenal stenting (*P* = 0.0042).

### Comparison of FT, *K*_a,r_ , and *P*_KA_ by stenosis site at each disease site

A comparative study was conducted to determine if there were differences in each parameter (FT, *K*_a,r_, and *P*_KA_) depending on the site of stenosis in each diseased site (Table S1, Supporting information). Stenosis sites were divided into upper, mid‐lower, and trans‐junction for the esophagus; above the pylorus, trans‐pylorus, and below the pylorus for gastroduodenum; and right side and left side for the colon. For esophageal stenting, there were no significant differences among the stenosis sites for any parameter. For gastroduodenal stenting, the FT was significantly shorter for the site above the pylorus (*P* = 0.0017) but there was no difference between the stenosis sites for *K*_a,r,_ and *P*_KA_. There were no significant differences between the stenosis sites for colonic stenting for any parameter.

## Discussion

The present study is a post hoc analysis of actual radiation dose data of fluoroscopy‐guided gastrointestinal procedures from the REX‐GI study, which is a nationwide, prospective observational study, regarding enteral metallic stent placement. We found that DRL values differed depending on the disease site. From the analysis of this study, DRLs for FT, the number of images, *K*_a,r_, and *P*_KA_ were significantly lower for esophageal stenting. Also, esophageal stenting had the shortest PT regardless of FT. Enteral metallic stent placement in the esophagus is a relatively simple procedure because of its linear structure, and multiple strictures in the esophagus are rarely compared with those in abdominal organs. For these reasons, enteral metallic stent placement in the esophagus had lower DRL values than gastroduodenal or colonic stenting. Therefore, we concluded that it is desirable to establish specific DRL values. The present study also showed that three disease sites—the esophagus, gastroduodenum, and colon—are sufficient for sub‐categorizing the DRLs for gastrointestinal stenting because further categorization by stenosis site within each disease site did not lead to a different RE. To our knowledge, there have been very few reports on DRLs for enteral metallic stent placement^14^; the UK Health Security Agency reported that the *P*_KA_ for esophageal stenting was 13 Gy cm^2^, which was slightly higher than that in this study (10.3 Gy cm^2^).^16^ However, no comprehensive study has reported a disease‐site‐specific analysis. Therefore, the present study is considered valuable because a relatively large number of patients who underwent enteral metallic stent placement were analyzed by specific disease sites.

A comparison of gastroduodenal stenting and colonic stenting showed significantly higher DRL values for FT in colonic stenting (*P* = 0.0248). Enteral metallic stent placement in the colon often requires more time to reach the lesion, especially on the right side of the colon. In addition, since most cases of colon stenting involve difficult insertion of the colonoscope due to the presence of severe adhesions, it tends to require a longer FT to simplify this insertion.^17^ Thus, FT during the procedure may strongly influence RE. In the case of difficult procedures, reducing the FT can be difficult, if necessary for treatment, because no dose limits apply to medical exposure. Regarding other ways to reduce RE, the use of real‐time monitoring techniques such as dose–area product meters or air kerma meters can help to optimize the reduction of RE during a procedure, which may allow the lowest possible radiation dose without compromising the quality of the procedure. Although there was no significant difference, *P*_KA_ tended to be higher for colonic stenting than for gastroduodenal stenting; the DRL values for *P*_KA_ were 48.4 Gy cm^2^ for the former and 36.6 Gy cm^2^ for the latter (*P* = 0.0759). On the other hand, *K*_a,r_ did not differ between gastroduodenal stenting and colonic stenting; the DRL values for *K*_a,r_ were 120 mGy for the former and 124 mGy for the latter (*P* = 1). This was because differences in the area exposed to radiation influence *P*_KA_, and colonic stenting requires a larger area than gastroduodenal stenting.

Thus, the present study showed that the characteristics of RE vary according to disease site, even among enteral metallic stent placements in the gastrointestinal tract. One of the reasons for the difficulty in setting DRLs is the wide distribution of disease sites, and one way to overcome this problem is by sub‐categorizing the procedures. In the Japan DRLs released in 2020, there was a twofold difference between chronic total occlusion (CTO) and non‐CTO in percutaneous coronary intervention (PCI), which was listed as a separate category.^6^ The present study indicates that it is desirable to set DRL values based on the disease site in the gastrointestinal tract for enteral metallic stent placement.

In recent years, various endoscopic procedures for diagnostic and therapeutic intervention have rapidly increased in popularity in gastrointestinal endoscopy units. The ICRP recommends that DRLs should be used to manage patient doses during both diagnostic and therapeutic procedures.^18^, ^19^, ^20^ The ICRP 135 publication recommends that all individuals involved in performing patient procedures with a risk of medical exposure be familiar with the DRL process as a tool for optimizing protection.^21^ DRLs vary from country to country because of differences in radiological practice and body size. Although it is desirable for each country to establish its own DRLs, there are currently few studies of DRLs for gastrointestinal stents on a national scale. Therefore, the comparison of DRLs in Japan with those in other countries remains an issue for future studies.

The REX‐GI study was launched to prospectively collect actual RE data and to establish DRLs for the following interventional procedures in gastrointestinal endoscopy units: ERCP, interventional endoscopic ultrasound (EUS), balloon‐assisted enteroscopy, enteral metallic stent placement, and enteral tube placement. The present study involved 23 medium‐ and large‐sized healthcare hospitals from all over Japan that perform sufficient numbers of procedures. The REX‐GI study had a larger sample size than previous studies that established DRLs in gastroenterology because this study prospectively registered more than 10 000 ERCPs, whereas the total number of ERCPs was approximately 1300 in the Japanese DRLs reported in 2020.^6^ In addition, this study comprised 523 cases of gastrointestinal enteral metallic stent placement. The ICRP 135 recommends using data from 20 to 30 facilities to set national DRLs, and a survey for a particular examination in a facility should usually involve the collection of data from at least 20 patients.^21^ Thus, the number of patients undergoing enteral metallic stent placement in this study would also be sufficient to establish optimal DRL values.

The current study had some limitations. The FT and number of imaging sessions, which are the basis for setting DRL values, may vary among institutions because of differences in technical skills and proficiency in the stenting procedure. This could potentially lead to variations in DRLs among different institutions. In our study, we considered these potential sources of variation by carefully selecting participating institutions of various levels—from university hospitals to municipal hospitals—to ensure a diverse sample. We also standardized the methodology for measuring and recording the FT and number of imaging sessions and trained all participating staff to ensure consistency in data collection. Overall, we took steps to minimize potential sources of variation and ensure the reliability and validity of our DRL values. In addition, we did not adjust for standard body size in this study.

## Conclusion

In conclusion, the present study provides significant data regarding actual RE during fluoroscopic enteral metallic stent placement in the gastrointestinal tract. These data indicate that it is desirable to set DRL values based on the disease site in the gastrointestinal tract for enteral metallic stent placement.

## Ethics statement

The study was registered with the UMIN Clinical Trials Registry (UMIN000036525, 1 May 2019). This study was conducted in accordance with the Declaration of Helsinki, and approval was obtained from each institutional review board. The requirement for informed consent was waived by the opt‐out method of each hospital website.

## Supporting information


**Table S1:** Comparison of FT, *K*_a,r_, and *P*_KA_ by stenosis site at each disease site.

## Data Availability

The data that support the findings of this study are available upon request from the corresponding author (Toshiyuki Yoshio).
