# Supplementary material for: Medical radiation exposure during gastrointestinal enteral metallic stent placement: Post hoc analysis of the REX‐GI study
Source: JGH Open. 2023 Dec 1;7(12):869–74. doi: 10.1002/jgh3.12993 (PMC10757487; doi:10.1002/jgh3.12993)
Supplement: Supplementary file 1 — Table S1: Comparison of FT, K a,r, and P KA by stenosis site at each disease site. [file JGH3-7-869-s001.docx]

Supplementary table: Comparison of FT, K_a, r_, and P_KA_ by stenosis site at each disease site

|  | Stenosis site | | | p value |
| --- | --- | --- | --- | --- |
| Esophageal stent | Upper n=16 | Mid-Lower n=59 | Transjunction n=38 |  |
| FT, median, min | 7.5 | 7.7 | 6.1 | 0.476 |
| K_a,r_, median, mGy | 28.6 | 30.6 | 26.2 | 0.565 |
| P_KA_, median, Gycm2 | 7.5 | 7.1 | 5.8 | 0.291 |
| Gastroduodenal stent | Above pylorus n=34 | Across pylorus n=66 | Below pylorus n=151 |  |
|  |  |  |  |  |
| FT, median, min | 7.4 | 9.0 | 11.1 | 0.002 |
| K_a,r_, median, mGy | 48.6 | 66.8 | 72.1 | 0.196 |
| P_KA_, median, Gycm2 | 14.4 | 18.6 | 20.8 | 0.099 |
| Colonic stent | Right side n=39 | Left side n=109 |  |  |
|  |  |  |  |  |
| FT, median, min | 13.5 | 12.0 |  | 0.396 |
| K_a,r_, median, mGy | 10.2 | 11.5 |  | 0.490 |
| P_KA_, median, Gycm2 | 22 | 21 |  | 0.682 |

FT, fluoroscopy time; K_a,r_, air kerma at the patient entrance reference point; P_KA_, air kerma-area product
